# Association Between History of Gestational Diabetes Mellitus and the Risk of Arthritis in Women

**DOI:** 10.3389/fpubh.2022.878845

**Published:** 2022-05-27

**Authors:** Yuanyuan Mao, Wenbin Hu, Bin Xia, Li Liu, Qin Liu

**Affiliations:** ^1^Suzhou Medical College of Soochow University, Suzhou, China; ^2^Department of Obstetrics and Gynecology, The First People's Hospital of Kunshan Affiliated With Jiangsu University, Suzhou, China; ^3^Department of Chronic and Noncommunicable Disease Control and Preventions, The Kunshan Center for Disease Control and Prevention, Suzhou, China

**Keywords:** arthritis, National Health and Nutrition Examination Survey, type 2 diabetes, metabolic syndrome, gestational diabetes mellitus

## Abstract

**Objective:**

The association between gestational diabetes mellitus (GDM) and the risk of arthritis has not been reported. GDM increases the risk of long-term complications including diabetes and metabolic syndrome that are positively associated with the risk of arthritis. This study aimed to explore the association between GDM and the risk of arthritis.

**Methods:**

Women (age ≥ 20 years) who had delivered at least one live birth were included from the 2007 to 2018 National Health and Nutrition Examination Survey cohort (*N* = 11,997). Patients who had a history of GDM and arthritis were identified by in-home interview. Subgroup analyses were conducted by arthritis types and status of obesity, current diabetes, metabolic syndrome, smoking, alcohol drinking, and physical activity.

**Results:**

GDM was associated with increased odds of arthritis [multivariable-adjusted odds ratio (95% confidence interval): 1.31 (1.06–1.62)], and the result was similar in sensitivity analysis with further adjustment for metabolic syndrome [1.30 (1.05–1.60)]. In subgroup analyses, GDM was associated with increased odds of osteoarthritis [1.47 (1.05–2.06)], while no association was observed with rheumatoid arthritis [1.04 (0.69–1.57)] and other types [1.26 (0.94–1.68)]. GDM was associated with increased odds of arthritis in women without metabolic syndrome [1.34 (1.00–1.78)] and diabetes [1.35 (1.03–1.76)], in obese individuals [1.64 (1.24–2.16)], current/former smokers [1.43 (1.05–1.95)], and current drinkers [1.76 (1.00–3.14)], and in individuals engaging in higher levels of physical activity [1.53 (1.06–2.20)].

**Conclusions:**

GDM was associated with increased odds of arthritis, and the association was independent of type 2 diabetes and metabolic syndrome.

## Introduction

Gestational diabetes mellitus (GDM) is diabetes that develops during pregnancy, and is currently the most common medical complication of pregnancy (1). The prevalence of GDM was estimated to be 7.6% in the US (2), and the overall weighted GDM prevalence in European countries was estimated at 10.9% (3). GDM increases the risk of long-term complications, including obesity, type 2 diabetes, metabolic syndrome, cancer, and cardiovascular disease (1, 4–7), and GDM provides unique opportunities for improving maternal health (8). Musculoskeletal conditions account for a significant proportion of non-communicable diseases contributing to disability adjusted life years, with osteoarthritis contributing most to this burden (9). The major arthritis-related disorders such as osteoarthritis and rheumatoid arthritis collectively make arthritis rank among the most common disabling health conditions (10). The global prevalence of rheumatoid arthritis was 460 per 100,000 population (11). The global age-standardized years lived with disability rate for osteoarthritis in 2017 was 118.8, an increase of 9.6% from 1990 (12).

Chronic inflammation may play a significant role in the development of arthritis-related disorders such as osteoarthritis and rheumatoid arthritis (13, 14). Inflammation and oxidative stress participate in the development of GDM and exert potentially harmful effects on the short and long-term maternal health (15). In addition, the long-term complications of GDM including obesity, type 2 diabetes, and metabolic syndrome are also positively associated with arthritis (16–20). However, the association between GDM and the risk of arthritis has not been reported in epidemiological studies. Based on the above-mentioned findings, we hypothesize that GDM is associated with increased odds of arthritis. In this study, we first explored the association between a history of GDM and the odds of arthritis in women, and then conducted stratified analyses to determine whether the association could still be observed in the absence of type 2 diabetes and metabolic syndrome. In addition, stratified analyses were also conducted by the modifiable risk factors for arthritis development, including smoking (21–23), alcohol drinking (24, 25), and low levels of physical activity (26, 27).

## Materials and Methods

### Study Populations

The National Health and Nutrition Examination Survey (NHANES) is a program of studies designed to assess the health and nutritional status of US adults and children. The survey examines a nationally representative sample of about 5,000 persons each year. We used data from six cycles of the NHANES cohort (2007/2008 to 2017/2018), as these cycles specifically provided information for a history of GDM.

The inclusion criteria are as follows: (1) women aged 20 years or older; (2) women with at least one live birth; (3) women responding to the questions regarding a history of GDM; and (4) women responding to the questions regarding arthritis. In addition, women who were diagnosed with diabetes or arthritis prior to a diagnosis of GDM were excluded. Finally, we included 11,997 women in this study.

### A History of GDM and Arthritis

The exposure for the analysis was the response to the question, “During your pregnancy, were you ever told by a doctor or other health professional that you had diabetes, sugar diabetes, or gestational diabetes?” and we considered women who answered yes to the above question as having a history of GDM (28, 29). The outcome for the analysis was arthritis, and patients with arthritis were identified with the questions of “doctor ever said you had arthritis?” and “which type of arthritis was it?”.

### Other Variables

According to the previous studies (23, 30, 31), the following covariates were included: age, race/ethnicity (Mexican–American, Other Hispanic, Non-Hispanic White, Non-Hispanic Black, Other Races), body mass index (BMI, under/normal weight: <25 kg/m^2^, overweight: 25 to <30 kg/m^2^, obesity: ≥30 kg/m^2^), education ( ≤ high school, some college or AA degree, ≥college graduate), annual family income (< $20,000, $20,000–$44,999, $45,000–$74,999, ≥$75,000), smoking (current smoker, former smoker, never smoker), alcohol drinking (g/day), and physical activity (vigorous/moderate recreational activities for at least 10 min continuously in a typical week). In addition, current diabetes and metabolic syndrome were also considered in stratified analyses. Current diabetes was defined using a self-reported diagnosis of diabetes outside pregnancy or, if diabetes was not previously diagnosed, by a hemoglobin A1c level ≥ 6.5%, a fasting plasma glucose level ≥ 126 mg/dL, or 2-h plasma glucose ≥ 200 mg/dL (32). Type 1 diabetes was defined as an onset age of self-reported diagnosis of diabetes <30 years and currently taking insulin (29). Any 3 of the 5 following metabolic-related disorders constitute diagnosis of metabolic syndrome (33): elevated blood pressure (≥130 mm Hg systolic blood pressure, ≥85 mm Hg diastolic blood pressure), elevated waist circumference (≥102 cm in men, ≥88 cm in women), reduced HDL-C (<40 mg/dL in men, <50 mg/dL in women), elevated triglycerides (≥150 mg/dL), and elevated fasting glucose (≥100 mg/dL).

### Statistical Analysis

The logistic regression was used to calculate the odds ratios (95% confidence interval) [OR (95% CI)] of arthritis for women with a GDM history compared with those without a history of GDM. We calculated three different logistic regression models. Model 1 was adjusted for demographic variables (age and race/ethnicity). Model 2 included the covariates of model 1 with additional adjustment for BMI and socioeconomic status (education and family income). Model 3 included the covariates of model 2 with additional adjustment for health behaviors (alcohol drinking, smoking, and physical activity). Stratified analyses were conducted by arthritis types (osteoarthritis, rheumatoid arthritis, and other types), current status of metabolic syndrome (yes, no), obesity (yes, no), and current diabetes (yes, no), smoking (never, current/former), alcohol drinking (yes, no), and physical activity (vigorous/ moderate recreational activities for at least 10 min continuously in a typical week: yes, no). Tests for interactions were performed by using cross-product terms of GDM with these stratified factors. In addition, because GDM was associated with lower HDL-C, and increased BMI, blood pressure, total cholesterol, triglycerides, and glucose (34), we also conducted a sensitivity analysis in which we further adjusted for metabolic syndrome to determine whether these metabolic-related disorders could account for the association between GDM and the risk of arthritis. New multi-year sample weight was computed by dividing the 2-year sample weights by 6 (six cycles of NHANES were included in this study). All analyses used sample weights, strata, and primary sampling units to account for the complex, multistage, stratified, and cluster-sampling design of NHANES. All analyses were conducted with Stata 12.0, and *P* ≤ 0.05 was considered statistically significant.

## Results

Among the 11,997 women included in this study, the weighted prevalence of GDM and arthritis was 8.00 and 34.72%, respectively. Women with a GDM history were more likely to be younger, engage in physical activity, and show higher prevalence of obesity, current diabetes (type 1 diabetes accounts for 2%), and metabolic syndrome. Annual family income and education levels differed significantly between women with a history of GDM and women without a history of GDM, while smoking status and alcohol drinking did not differ significantly between the two groups. Detailed characteristics of the participants are shown in Tables 1, 2. The associations between the characteristics of the participants and arthritis are shown in Table 3, and all these characteristics were associated with arthritis.

**Table 1:** Characteristics of the 2007–2018 NHANES adults according to the presence or absence of a history of gestational diabetes mellitus (GDM).

**Variables**	**Women with GDM**	**Women without GDM**	***P^***a***^***
	**(928)**	**(11,069)**	
Age [years, mean (SD)]	45.45 (12.31)	53.47 (16.76)	<0.01
Race/ethnicity (%)			<0.01
Mexican American	21.77	15.78	
Other Hispanic	11.42	11.66	
Non-Hispanic White	35.78	40.77	
Non-Hispanic Black	17.78	22.12	
Other Race	13.25	9.66	
Annual family income (%)			<0.01
< $20,000	20.74	26.64	
$20,000–$34,999	33.37	33.71	
$35,000–$74,999	18.83	17.58	
≥$75,000	27.06	22.07	
Education (%)			<0.01
≤ high school	43.64	50.14	
Some college or AA degree	35.99	30.63	
≥College graduate	20.37	19.23	
Vigorous/moderate recreational activities for at least 10 min continuously in a typical week (%)	44.29	40.85	0.04
Smoking (%)			0.34
Current smoker	18.86	17.45	
Former smoker	18.00	19.64	
Never smoker	63.15	62.91	
Alcohol (g/day)	4.21	4.04	0.70
Obesity (%)	56.06	43.05	<0.01
Current diabetes (%)	35.56	18.21	<0.01
Metabolic syndrome (%)	49.78	40.18	<0.01

*M, Mean values; SD, standard deviation*.

*^a^ t-test was performed for continuous variables, and Chi-square test was performed for categorical variables*.

**Table 2:** Characteristics of the 2007–2018 NHANES adults according to the presence or absence of arthritis.

**Variables**	**Women with arthritis**	**Women without arthritis**	***P^***a***^***
	**(4,293)**	**(7,704)**	
Age [years, mean (SD)]	62.53 (12.22)	47.46 (15.82)	<0.01
Race/ethnicity (%)			<0.01
Mexican American	11.95	18.64	
Other Hispanic	10.65	12.20	
Non-Hispanic White	48.26	35.99	
Non-Hispanic Black	22.36	21.47	
Other Race	6.78	11.70	
Annual family income (%)			<0.01
< $20,000	33.10	22.32	
$20,000–$34,999	33.47	33.80	
$35,000–$74,999	15.97	18.63	
≥$75,000	17.45	25.25	
Education (%)			<0.01
≤ High school	54.27	47.06	
Some college or AA degree	31.06	31.03	
≥College graduate	14.67	21.92	
Vigorous/ moderate recreational activities for at least 10 min continuously in a typical week (%)	31.10	40.49	<0.01
Smoking (%)			<0.01
Current smoker	18.57	17.00	
Former smoker	26.42	15.67	
Never smoker	55.02	67.33	
Alcohol (g/day)	3.67 (12.83)	4.27 (13.26)	0.02
Obesity (%)	53.02	39.10	<0.01
Current diabetes (%)	29.54	13.99	<0.01
Metabolic syndrome (%)	51.46	35.05	<0.01

*M, Mean values; SD, standard deviation*.

*^a^t-test was performed for continuous variables, and Chi-square test was performed for categorical variables*.

**Table 3:** Association between population characteristics and arthritis.

**Characteristics**	**Odds ratio (95% confidence**	***P^***a***^***
	**interval) of arthritis**	
Age	1.07 (1.06–1.08)	<0.01
Race/ethnicity		
Mexican American	1.00	
Other Hispanic	1.41 (1.20–1.65)	<0.01
Non-Hispanic White	2.54 (2.21–2.91)	<0.01
Non-Hispanic Black	1.91 (1.67–2.19)	<0.01
Other Race	1.45 (1.14–1.84)	<0.01
Annual family income		
< $20,000	1.00	
$20,000–$34,999	0.76 (0.67–0.85)	<0.01
$35,000–$74,999	0.65 (0.55–0.75)	<0.01
≥$75,000	0.52 (0.44–0.61)	<0.01
Education		
≤ High school	1.00	
Some college or AA degree	0.90 (0.79–1.02)	0.11
≥College graduate	0.58 (0.50–0.67)	<0.01
Vigorous/ moderate recreational activities for at least 10 min continuously in a typical week	1.41 (1.27–1.58)	<0.01
Smoking		
Never smoker	1.00	
Former smoker	1.95 (1.68–2.27)	<0.01
Current smoker	1.37 (1.22–1.54)	<0.01
Alcohol (g/day)	0.99 (0.98–1.00)	<0.01
Obesity	2.09 (1.84–2.36)	<0.01
Current diabetes	2.91 (2.61–3.25)	<0.01
Metabolic syndrome	2.08 (1.88–2.31)	<0.01

^a^*The P values for significance tests*.

**Figure 1:**
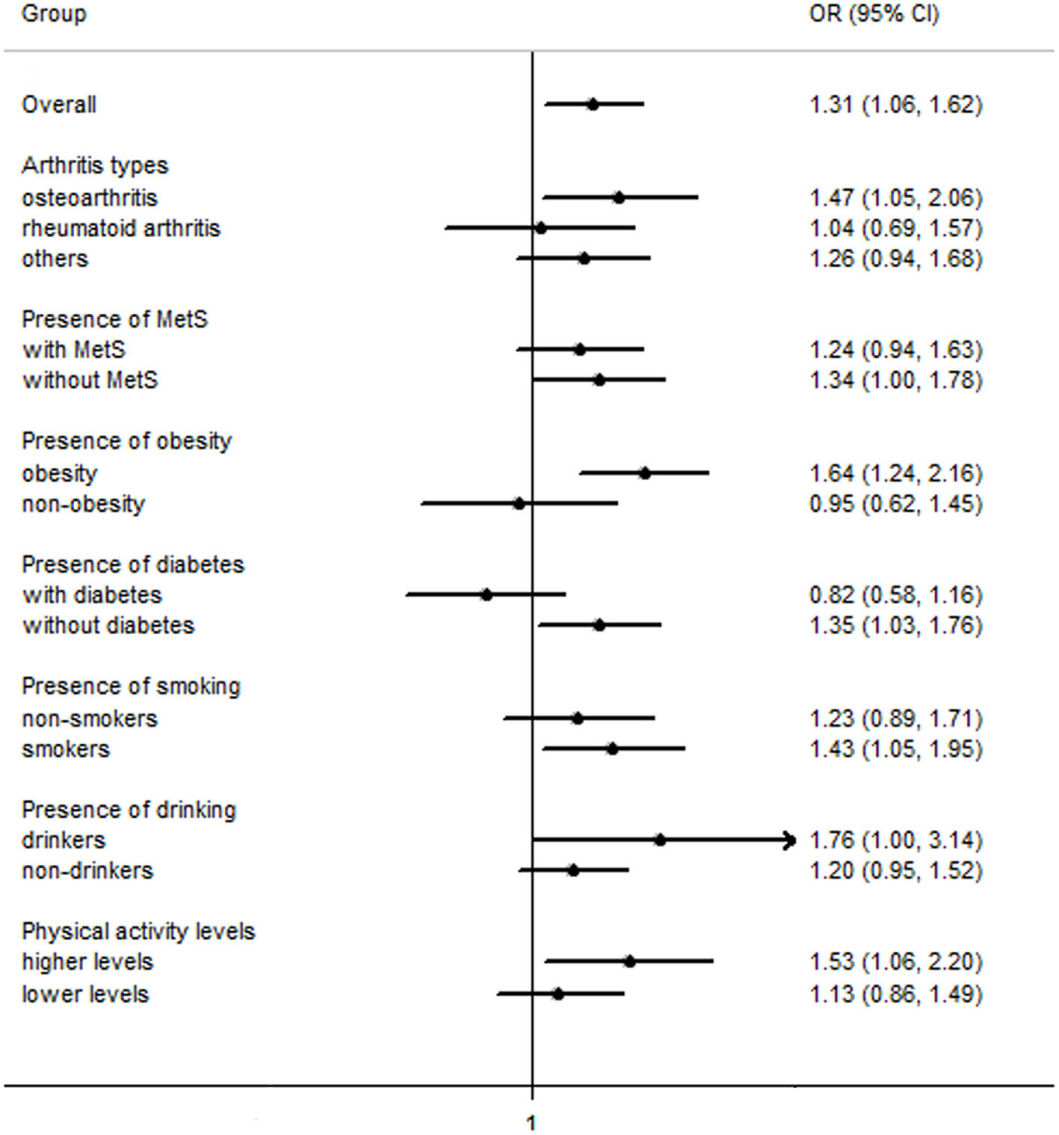
Odds ratios of arthritis for women with a history of gestational diabetes mellitus compared with those without a history of gestational diabetes mellitus. MetS, metabolic syndrome; OR (95% CI), Odds ratio (95% confidence interval).

Overall, the findings on the association between a history of GDM and the odds of arthritis were similar across the three statistical models, while the observed association was attenuated slightly in model 2 and model 3. In model 3, a history of GDM was associated with increased odds of arthritis [OR (95% CI): 1.31 (1.06–1.62), *P* < 0.05], and the result was similar in sensitivity analysis with further adjustment for metabolic syndrome [1.30 (1.05–1.60)]. In subgroup analyses by arthritis types, a history of GDM was associated with increased odds of osteoarthritis [1.47 (1.05–2.06)], while no association was observed with rheumatoid arthritis [1.04 (0.69–1.57)] and other types [1.26 (0.94–1.68)] (Table 4, Figure 1).

**Table 4:** Odds ratios of arthritis for women with a history of gestational diabetes mellitus compared with those without a history of gestational diabetes mellitus.

**Groups**	**Odds ratios (95% confidence intervals)**				
	**Cases of arthritis/N**	**Model 1**	**Model 2**	**Model 3**	***P*_for interaction_**
Overall	4,293/11,997	1.46 (1.18–1.80)**	1.32 (1.06–1.64)*	1.31 (1.06–1.62)*	
Arthritis types
Osteoarthritis	1,798/11,997	1.66 (1.22–2.29)**	1.46 (1.05–2.03)*	1.47 (1.05–2.06)*	
Rheumatoid arthritis	834/11,997	1.17 (0.77–1.77)	1.11 (0.75–1.63)	1.04 (0.69–1.57)	
Others	1,661/11,997	1.32 (0.99–1.76)	1.25 (0.93–1.67)	1.26 (0.94–1.68)	
Metabolic syndrome					0.12
Yes	2,209/4,909	1.29 (0.99–1.69)	1.18 (0.89–1.57)	1.24 (0.94–1.63)	
No	2,084/7,088	1.42 (1.07–1.88)*	1.39 (1.05–1.85)*	1.34 (1.00–1.78)*	
Obesity					0.05
Yes	2,244/5,233	1.61 (1.24–2.10)**	1.65 (1.24–2.19)**	1.64 (1.24–2.16)**	
No	1,988/6,643	0.95 (0.63–1.43)	0.93 (0.61–1.41)	0.95 (0.62–1.45)	
Current diabetes					<0.01
Yes	1,268/2,346	0.86 (0.62–1.20)	0.83 (0.60–1.15)	0.82 (0.58–1.16)	
No	3,025/9,651	1.39 (1.06–1.83)*	1.34 (1.01–1.76)*	1.35 (1.03–1.76)*	
Smoking					0.59
Never	2,362/7,549	1.40 (1.04–1.89)*	1.25 (0.91–1.73)	1.23 (0.89–1.71)	
Current/former	1,931/4,448	1.59 (1.18–2.14)**	1.42 (1.05–1.94)*	1.43 (1.05–1.95)*	
Current drinker					0.42
Yes	3,355/9,152	1.81 (1.05–3.13)*	1.68 (0.98–2.87)	1.76 (1.00–3.14)*	
No	938/2,845	1.35 (1.09–1.67)**	1.22 (0.97–1.53)	1.20 (0.95–1.52)	
Physical activity^a^					0.63
Yes	2,862/7,064	1.63 (1.15–2.32)**	1.51 (1.07–2.13)*	1.53 (1.06–2.20)*	
No	1,431/4,933	1.32 (1.00–1.74)*	1.16 (0.87–1.53)	1.13 (0.86–1.49)	

*P <0.05, **P <0.01.

*Model 1: adjusted for age and race/ethnicity.*

*Model 2: adjusted for covariates in model 1 and body mass index, education, and annual family income.*

*Model 3: adjusted for covariates in model 2 and alcohol drinking, smoking, and recreational physical activity.*

*^a^Vigorous/moderate recreational activities for at least 10 min continuously in a typical week*.

In stratified analyses by current status of metabolic syndrome, diabetes, obesity and physical activity, a history of GDM was associated with increased odds of arthritis in obese women [1.64 (1.24–2.16)] and in women without metabolic syndrome [1.34 (1.00–1.78)] and current diabetes [1.35 (1.03–1.76)]. In stratified analyses by status of smoking, alcohol drinking and physical activity, a history of GDM was associated with increased odds of arthritis in current/former smokers [1.43 (1.05–1.95)], current drinkers [1.76 (1.00–3.14)], and individuals engaging in higher levels of physical activity [1.53 (1.06–2.20)]. The interactions between a history of GDM and obesity (*P* = 0.05) and current diabetes (*P* < 0.01) were significant. However, the interactions with metabolic syndrome (*P* = 0.12), smoking (*P* = 0.59), alcohol drinking (*P* = 0.42), and physical activity (*P* = 0.63) were not significant (Table 4), respectively, which may arise from the relatively wide range of 95% CIs or the possibility that these variables are independent of the history of GDM.

## Discussion

To our knowledge, this is the first epidemiological study to explore the association between GDM and the risk of arthritis. In this national survey cohort of 11,997 women, women with a history of GDM tended to have increased odds of arthritis, and the finding was robust even after accounting for metabolic syndrome that is potentially related to subsequent arthritis. Importantly, women with a history of GDM in whom metabolic syndrome and diabetes does not develop still have increased odds of arthritis. In stratified analyses, the association between a history of GDM and arthritis was observed in smokers, alcohol drinkers, and women engaging in higher levels of physical activity. In addition, GDM was significantly associated with increased odds of osteoarthritis, while no significant association was found with rheumatoid arthritis and other types.

Several potential reasons may explain the association between GDM and the risk of arthritis. First, women with a history of GDM were found to have a nearly 10-fold higher risk of developing type 2 diabetes than healthy controls [relative risk (95% CI): 9.51 (7.14–12.67)] (4), and a previous meta-analysis showed that type 2 diabetes was associated with increased odds of arthritis [OR (95%): 1.45 (1.18–1.78)] (20). Furthermore, patients with diabetes mellitus had 2.18 [95% (1.12–4.24)] times the odds of having osteoarthritis (35), which is the most common type of arthritis (13). Second, a recent meta-analysis found that the OR (95% CI) for metabolic syndrome was 3.45 (2.80–4.25) in women with a history of GDM compared to women without a history of GDM (5), and metabolic syndrome [OR (95% CI): 1.42 (1.16–1.73)], hypertension [1.70 (1.41–2.05), and hyperglycemia [1.23 (1.05–1.42)] were all positively associated with odds of osteoarthritis (19). Third, women with a history of GDM have significantly higher BMI (1.54 kg/m^2^, 95% CI: 1.32 to 2.46) (34). Compared to normal weight subjects, overweight and obese subjects had 15% (95%CI: 1.03–1.29) and 31% (1.12–1.53) higher odds of rheumatoid arthritis (36), respectively, and the associations were stronger with osteoarthritis [overweight: 2.45 (1.88–3.20), obesity: 4.55 (2.90–7.13)] (18). These findings are consistent with those from our study in which a history of GDM was associated with odds of osteoarthritis but not rheumatoid arthritis. In addition, the association between a history of GDM and odds of arthritis was only observed in subjects with obesity [1.64 (1.24–2.16)], and the association was attenuated in model 2 also adjusting for BMI [1.32 (1.06–1.64)]. These findings suggested that BMI may partially account for the observed association between GDM and the odds of arthritis. However, the association between a history of GDM and the odds of arthritis remained significant in women without metabolic syndrome [1.34 (1.00–1.78)] and current diabetes [1.35 (1.03–1.76)], and a similar result was found in sensitivity analysis with further adjustment for metabolic syndrome [1.30 (1.05–1.60)]. These findings suggested the observed association between a history of GDM and the odds of arthritis was independent of type 2 diabetes and metabolic syndrome. These findings are comparable with those from previous studies in which the long-term risk for cardiovascular disease associated with GDM was not dependent upon intercurrent type 2 diabetes (7), but maybe explained partly by BMI (28).

Results from this study showed that a history of GDM was significantly associated with increased odds of arthritis among smokers, alcohol drinkers, and women engaging in higher levels of physical activity. While smoking was inversely associated with the risk of osteoarthritis in both observational studies (22) and Mendelian randomization studies (21, 37), a positive association was found between smoking and the risk of rheumatoid arthritis in both observational studies (38) and a Mendelian randomization study (39). These results indicate that the effects of smoking on arthritis may differ by different clinical subtypes of arthritis. Low to moderate alcohol consumption was found inversely associated with the development of both osteoarthritis (24) and rheumatoid arthritis (25), while a positive association was also found between alcohol consumption and osteoarthritis prevalence in the Korean NHANES assessed by the alcohol use disorders identification test (40). Findings on the association between physical activity and arthritis are conflicting. While physical activity was found to be inversely associated with arthritis in observational studies (26, 27), physical activity may constitute an important risk factor for arthritis progression prediction with a machine learning approach (41). In this study, physical activity was also found positively associated with prevalence of arthritis [1.41 (1.27–1.58)] (Table 3). These findings indicate that the association between physical activity and arthritis may differ by clinical subtypes of arthritis, and intensity and measurement methods of physical activity (26, 27, 41), which need to be confirmed further. In summary, the findings available on the associations between smoking, alcohol drinking, and physical activity and arthritis remain contradictory, and the potential interactions between a history of GDM and these life-style factors on the risk of arthritis deserve to the confirmed further.

There are several limitations in this study. First, the causality cannot be determined because this is a cross-sectional study, and the causality should be confirmed further in prospective cohort studies. Second, a GDM history and arthritis diagnosis were based on self-report and misclassification could be of concern. However, data from the NHANES are considered to be valid to assess the prevalence of GDM and arthritis in the general population (10, 28, 29), and misclassification of patients with undiagnosed arthritis and GDM as healthy controls could have weakened the association.

In summary, a history of GDM was associated with increased odds of arthritis in this nationally representative cohort, and the association was independent of metabolic syndrome and type 2 diabetes. The association between a history of GDM and arthritis was observed in smokers, alcohol drinkers, and women engaging in higher levels of physical activity. The causality should be confirmed further in prospective cohort studies.

## Code Availability

Analytic code will be made available from the corresponding author.

## Data Availability Statement

The original contributions presented in the study are included in the article/supplementary files, further inquiries can be directed to the corresponding author/s.

## Ethics Statement

The studies involving human participants were reviewed and approved by National Center for Health Statistics Research Ethics Review Board. The patients/participants provided their written informed consent to participate in this study.

## Author Contributions

YM and QL designed the study. WH conducted the statistical analysis. YM, BX, LL, and QL drafted the manuscript. QL made critical revisions. All authors contributed to the article and approved the submitted version.

## Funding

The authors received support from the Maternal and Child Health Research Project of Jiangsu Province (No. F201720) and the Development Science and Technology Project of Kunshan (No. KS1646).

## Conflict of Interest

The authors declare that the research was conducted in the absence of any commercial or financial relationships that could be construed as a potential conflict of interest.

## Publisher's Note

All claims expressed in this article are solely those of the authors and do not necessarily represent those of their affiliated organizations, or those of the publisher, the editors and the reviewers. Any product that may be evaluated in this article, or claim that may be made by its manufacturer, is not guaranteed or endorsed by the publisher.
